# Influence of *Pleurotus eryngii* Protein on Myofibrillar Protein Gelation and Application in Chicken Mince Products

**DOI:** 10.3390/foods14050752

**Published:** 2025-02-23

**Authors:** Li Zhao, Weiwei Yan, Zheming Wang, Jiaman Wu, Liang Li, Shaojun Yun, Wenfei Zhao, Cuiping Feng

**Affiliations:** College of Food Science and Engineering, Shanxi Agricultural University, Jinzhong 030801, China

**Keywords:** *Pleurotus eryngii*, myofibrillar protein, composite gel, chicken mince, quality

## Abstract

*Pleurotus eryngii* is rich in essential proteins, and is recognized for its medicinal and nutritional attributes. This study investigated the effects of *P. eryngii* protein (PEP) incorporation (0–7%) on the gel characteristics of chicken myofibrillar protein (MP) to develop a chicken mince product, providing theoretical supports for the development of functional restructured meat products. PEP incorporation improved the rheological and textual properties of the composite gels, enhancing hydrophobic interaction and disulfide bond formation, and thereby strengthening the gel characteristics. The optimal chicken mince quality was achieved with the incorporation of 3% *P. eryngii* powder. These findings highlight that PEP contributes to the MP gel functionality by modifying the gel structure and strengthening the molecular bonds, laying a foundation for applying PEP in food processing.

## 1. Introduction

Processed meat products are highly favored by consumers for their soft texture, elasticity, and ease of mastication and digestion [1]. These unique organoleptic properties are formed under the combined effects of the stability of the gel structure, matrix composition, and interaction between water molecules, which comprehensively influence the overall texture and quality of the meat products [2]. Myofibrillar protein (MP) plays a pivotal role in constructing the gel framework structure of meat products. As one of the most crucial proteins in meat, MP accounts for approximately 60% of the total muscle protein. Its family members, such as actin, myosin, and actomyosin, can interact with each other to form a viscoelastic three-dimensional gel network [3]. This network structure provides the necessary binding strength for meat products, effectively immobilizes water molecules and fat particles, and is of great significance for maintaining the structural integrity and water retention of meat products [4].

However, during processing, storage, and transport, single-gel meat products are extremely susceptible to the negative impacts of various external factors. These factors can induce variations in the physical, chemical, and structural properties of MP, thereby reducing the strength, elasticity, water-holding capacity (WHC), and slicing properties of the gel and ultimately diminishing the overall quality of the product [5]. Therefore, optimizing the processing performance of MP is of great importance for enhancing the quality of meat products. In meat processing, it is generally imperative to refine the processing techniques and/or incorporate other ingredients to augment the WHC of meat products, which is pivotal for elevating their visual appeal and overall quality. The ultimate aim is to facilitate the formation of a stable and robust MP network [6]. For example, ultrasonic-assisted sodium bicarbonate treatment can increase the compactness of the reduced-salt MP gel structure and, at the same time, enhance the WHC and textural characteristics [7]. The pH value significantly affects the gel properties of *Flammulina velutipes* polysaccharide (FVP)-MP composite gel. With an increase in the pH value, the WHC, oil-holding capacity, hardness, and elasticity of the FVP-MP gels increase significantly [8]. The addition of okra polysaccharide enhanced the interactions between MPs, resulting in a denser intermolecular gel network structure [9]. A study used chicken fat, refined palm oil (RPO), and palm stearin to emulsify the extracted chicken MP. The results showed that RPO may be the best lipid source for emulsification of chicken protein due to its high WHC and excellent thermal and rheological properties [10]. In recent years, incorporating non-meat proteins into meat products has emerged as a strategy for fat replacement, simultaneously enhancing the quality of these products [11]. The addition of soy protein isolate was found to result in smoother, denser, and whiter surimi gels, with a whiteness value reaching 61.49. These gels also exhibited excellent textural properties, a high WHC of 85.67%, and enhanced structural integrity [12]. The addition of faba bean protein isolate may improve the rheological properties of the MP gels and the functional properties of pork low-fat model sausages, including WHC and textural properties [13]. Therefore, non-meat proteins have exhibited outstanding performance in improving the properties of meat products with great application potential.

*Pleurotus eryngii* is an edible fungus known for its high contents of protein, polysaccharides, magnesium, calcium, and other vital nutrients, and is a special food with medicinal properties [14]. The protein derived from *P. eryngii* (PEP) is nutritionally equivalent to meat, egg, and soybean protein, and can meet the nutritional requirements of the human body. Therefore, PEP has significant potential as a functional ingredient in the food industry for its rich nutritional profile and a variety of functional attributes, such as emulsification, gelation, foaming, and flavor-binding capabilities [15]. Previous research has mostly focused on the structural changes of PEP and its biological activities under different treatments. However, the specific impact of PEP on the MP gel and the underlying mechanisms remain to be comprehensively explored.

In this study, we first added PEP to MP to enhance its gelation properties, and comprehensively analyzed the composite gel system, including WHC, cooking yield, thermal stability, structural characteristics, surface hydrophobic properties, and rheological properties. Furthermore, we investigated the chemical bonding forces, covalent interactions, storage modulus, and microstructure of the composite gel to elucidate the underlying mechanism by which PEP affects the gelation performance of MP. The results demonstrated that PEP could optimize the performance of the MP–PEP composite gel. Based on the findings, we developed a chicken mince product with the incorporation of *P. eryngii* powder. Analysis of the product indicated that the incorporation of PEP facilitates more precise regulation of the gelation properties of MP, demonstrating the promising prospect and potential for the practical application of PEP in the food industry.

## 2. Materials and Methods

### 2.1. Materials and Reagents

*P. eryngii* was provided by the Shanxi Engineering Research Center of Edible Fungi (Taigu, China). The *P. eryngii* utilized in the experiments were harvested at nearly the same time and grown under identical environmental conditions. During transportation and storage, they were kept in a dry and low-temperature environment to mitigate quality alterations. The contents of ash, fat, protein, and total sugars in *P. eryngii* were 3.49%, 4.37%, 21.63%, and 34.39%, respectively. Chicken breasts were sourced from the same supplier and the same breeding batch. After purchase, they were processed promptly to prevent a decrease in freshness caused by prolonged storage. On the day of the experiment, the appearance, odor, and texture of the chicken breasts were re-inspected. Only when it was ensured that there were no off-odors, and no signs of spoilage, were subsequent experiments conducted. Na_2_EDTA, Tris, glycine, and β-mercaptoethanol were purchased from Beijing Solarbio Science & Technology Co., Ltd. (Beijing, China). All other chemicals were of analytical reagent grade.

### 2.2. Extraction of PEP

The PEP was extracted as described by a previous study [16], with minor modifications. Briefly, *P. eryngii* powder was mixed with deionized water at a ratio of 1:55 (*w*/*v*). The pH of the mixture was adjusted to 12.0 using 1 M NaOH solution. After stirring at 50 °C for 2.5 h, the mixture was centrifuged at 1700× *g* for 15 min to separate the supernatant. Then, the pH of the supernatant was adjusted to 3.6 with 0.01 M HCl solution and left standing for 1 h. Subsequently, a second centrifugation at 1700× *g* for 10 min was performed to precipitate the protein. The precipitated protein was re-dissolved in deionized water and neutralized to pH 7.0 using 0.01 M NaOH solution. Finally, the protein solution was freeze-dried to obtain PEP. The extracted samples were stored at −20 °C for further use.

### 2.3. Extraction of MP

The extraction of MP was conducted following a method reported in the literature [17], with minor modifications. After removal of the visible fat and connective tissues, the chicken breast was minced into a meat paste. A 5 g portion of the chicken meat paste was added to a separation buffer with a volume four times that of the chicken meat paste (*w*:*v*). The separation buffer consisted of 0.1 M KCl, 4.3 mM MgCl_2_, 25.2 mM Na_2_HPO_4_, and 1 mM EDTA with a pH of 7. The mixture was homogenized at 8000× *g* for 30 s and then centrifuged at 4000× *g* for 15 min at 2 °C. The precipitate was retained. The procedure was repeated twice by adding the separation buffer with four times the volume each time. Finally, the precipitate was added with a 0.1 M NaCl solution with a volume eight times that of the precipitate. The mixture was homogenized and centrifuged three times under the same conditions, with an additional 30 s homogenization after the last centrifugation. The mixture was filtered through gauze, and the filtrate was adjusted to pH 6.2 with 0.1 M HCl and centrifuged at 4000× *g* for 15 min at 2 °C. The supernatant was discarded to obtain MP. The concentration of the extracted protein was determined using the biuret method with bovine serum albumin (BSA) as the standard. The extracted chicken MP was stored at 4 °C and used within 48 h to ensure freshness and quality for subsequent applications.

### 2.4. Preparation of the MP–PEP Composite Gels

First, 1%, 3%, 5%, and 7% of PEP were mixed with MP, respectively, and dissolved in 50 mM phosphate buffer solution containing 0.6 M NaCl at pH 6 to ensure the total protein concentration of 60 mg/mL. The mixed solution was directly used for rheological measurement and thermal denaturation analysis. The mixed solution containing 60 mg/mL MP was heated in a water bath at 90 °C for 1 h, then quickly removed and cooled in ice water, and placed in a refrigerator at 4 °C for 12 h. The prepared gel was equilibrated at room temperature for 30 min before the determination of the gel properties, such as gel strength, WHC, and cooking yield.

### 2.5. Determination of the Composite Gel Properties

#### 2.5.1. WHC and Cooking Yield Measurement

The WHC of the composite gels was tested according to the previously published method [18]. The samples were weighed (*M1*) and loaded onto a triple layered filter paper before being put into 50 mL centrifuge tubes. These samples were subjected to centrifugation at a speed of 2700× *g* for 15 min at 4 °C. After centrifugation, the gel samples were immediately re-weighed to obtain the new mass (*M*2). The calculation formula is as follows:

$$WHC\left(\%\right)=\left(\frac{M2}{M1}\right)\times 100$$

The cooking yield, which represents the efficiency of the heat-induced treatment process, is determined by the weight ratio of the sample before and after the treatment. It was calculated using the following formula:

$$Cooking\;Yield\left(\%\right)=\left(\frac{W1}{W0}\right)\times 100$$

where *W*0 is the initial weight of the samples and *W*1 is the weight of the sample after cooking.

#### 2.5.2. Gel Textural Properties

The gel strength of the composite protein gels was determined using a texture analyzer (CT3 4500, Brookfield Co., Ltd., Middleboro, MA, USA). A P/0.5 probe and a compression distance of 4 mm were used for the test. The program parameters were as follows: pre-test speed: 60 mm/min; post-test speed: 300 mm/min; trigger force: 5.0 g.

#### 2.5.3. Determination of Thermal Denaturation

First, 10 mg of freeze-dried gel sample was introduced into an aluminum crucible for thermal analysis. The differential scanning calorimeter (Q20, TA Corp., Newcastle, DE, USA) was utilized to incrementally raise the temperature from 20 °C to 140 °C at a consistent rate of 10 °C/min. The denaturation temperature (Td) of the samples, which is the temperature at which the protein structure begins to unfold, or the gel matrix starts to break down, was meticulously recorded.

#### 2.5.4. Surface Hydrophobicity

The surface hydrophobicity of the MP–PEP was evaluated using the ANS (8-Anilino-1-naphthalenesulfonic acid) fluorescence probe [19]. In brief, the MP–PEP mixed protein samples were diluted to a protein concentration of 0.5 mg/mL with a 50 mM phosphate buffer solution (pH 6). A 4 mL sample solution was taken and 20 μL of the fluorescent probe (8 mM ANS) was added. The mixture was vortexed and allowed to react statically for 2 min. The fluorescence intensity was measured using a Lumina fluorescence spectrophotometer (Thermo, Waltham, MA, USA) with an excitation wavelength set at 375 nm and scanned in the wavelength range from 390 to 650 nm. The slit width was set at 5 nm.

#### 2.5.5. Rheological Properties

Dynamic rheological measurements of the MP–PEP composite gels were conducted using an MCR102 rheometer (Anton Paar Co., Ltd., Sydney, Australia) to determine the viscoelastic dynamics [20]. A 2.5 mL sample solution was loaded onto a parallel plate with a slit distance of 1 mm and heated from 20 °C to 80 °C at a heating rate of 2 °C/min. Prior to heating, the exposed samples were sealed with silicone oil to prevent water evaporation from the protein solution during the heating process. The changes in the storage modulus (G′) were continuously recorded.

#### 2.5.6. Intermolecular Force Measurement

The molecular forces of the samples were determined following a method reported in the literature [21], with slight modifications. Briefly, gel samples (1 g) were dissolved in 10 mL of different denaturing solutions, including 0.05 M NaCl (F1), 0.6 M NaCl (F2), 0.6 M NaCl + 1.5 M urea (F3), 0.6 M NaCl + 8 M urea (F4), 0.6 M NaCl + 8 M urea + 0.5 M β-mercaptoethanol (F5). The gel dispersions were homogenized at 8000× *g* for 1 min and left at 4 °C for 2 h. Then, the supernatant was centrifuged at 4 °C and 10,000× *g* for 15 min, and the protein content of the supernatant was determined using Lowry’s method. The differences between F2 and F1, F3 and F2, F4 and F3, and F5 and F4 represent the presence of ionic bonds, hydrogen bonds, hydrophobic interactions, and disulfide bonds, respectively.

#### 2.5.7. Determination of the Particle Size and Zeta Potential

The particle size and zeta potential of the samples were determined using a Zetasizer nano ZS90 Malvern dynamic light scattering instrument (Malvern Panalytical Ltd., Malvern, UK). Briefly, the MP–PEP mixed protein samples were diluted with distilled water to 0.1 mg/mL, respectively. The parameters of the potential test program were set at a scattering angle of 90° and an equilibrium time of 120 s. All measurements were carried out at 25 °C [22].

#### 2.5.8. FT-IR and Fluorescence Spectroscopy Analysis

The composite gels were lyophilized using a freeze dryer (SZFD-3A, Shanghai Shun System Instrument Manufacturing Co., Ltd., Shanghai, China). The sample powder was mixed with KBr in a ratio of 1:100 for tablet pressing. FT-IR spectra were obtained using an IR spectrometer (Tensor 27, Bruker, Karlsruhe, Germany) in the range of 4000–400 cm^−1^ with a resolution of 4 cm^−1^ [23].

Fluorescence spectral analysis of the samples was carried out at 298 K using a Lumina fluorescence spectrofluorometer (Thermo, USA). The MP–PEP mixed protein solution was diluted to 0.1 mg/mL using 50 mM phosphate buffer solution (pH 6). The excitation wavelength was set at 285 nm with a slit width of 5 nm, and the emission spectrum was recorded in the range of 300–480 nm.

#### 2.5.9. Scanning Electron Microscopy (SEM)

The MP–PEP gel samples were treated with a 2.5% glutaraldehyde solution in 0.1 M phosphate buffer (pH 7.0) for 2 h. Subsequently, the samples were dehydrated with ethanol and then subjected to freeze-drying. The lyophilized samples were coated with a thin layer of gold via sputtering and then subjected to scanning electron microscopy (JSM-7500F, JEOL, Tokyo, Japan) at an accelerating voltage of 10 kV.

### 2.6. Preparation of the P. eryngii Chicken Mince

The excess fat and connective tissues of the fresh chicken breast were removed and minced into a meat paste. The meat paste was mixed with 1%, 3%, 5%, and 7% *P. eryngii* powder, respectively (the control group had no incorporation of the *P. eryngii* powder). Then, 0.2% compound phosphate and 40% water (both based on the mass of the meat paste) were added successively. After chopping and mixing for 3 min, the mixture was marinated at 4 °C for 2 h, packaged, and stored at −20 °C for future utilization.

### 2.7. Quality Determination of the P. eryngii Chicken Mince

#### 2.7.1. Rheological Property and WHC Measurements

Rheological properties of the *P. eryngii* chicken mince was assessed using a previously reported method with slight modifications [24]. A PP50 flat cylindrical probe was utilized for this test, which was conducted at a temperature of 25 °C. The range of linear viscoelastic behavior of the sample can be determined by strain scanning analysis. Within this linear viscoelastic domain, a frequency sweep was executed with frequencies ranging from 1 Hz to 16 Hz.

The WHC of the chicken mince was determined by the method described in Section 2.5.1.

#### 2.7.2. Color, Texture, and Sensory Evaluation

The mince sample was boiled at 85 °C for 25 min, followed by cooling. Subsequently, the color, texture, and sensory properties were measured. To measure the L*, a*, and b* values, a colorimeter (CM-5, Konica Minolta Inc., Tokyo, Japan) was utilized. The instrument was calibrated using a standard blackboard and whiteboard before the test (whiteboard L* = 75.96, a* = −1.44, b* = 3.22). The whiteness (W) of samples was calculated using the following equation:

$$W=100-\sqrt{{\left(100-L^{*}\right)}^{2}+{a^{*}}^{2}+{b^{*}}^{2}}$$

The procedure for texture determination was as follows. A P/50 sensor was employed, and the pre-test velocity was set at 2 mm/s, with a testing velocity of 1 mm/s and a post-test velocity of 2 mm/s. The force gauge should be calibrated within a range of 100 N, with the target distance set at 5 mm.

Ten professional evaluators were selected to form a sensory evaluation panel. The evaluation criteria and precautions for sensory indicators were clearly defined. Sensory scoring of the quality of the *P. eryngii* chicken mince was carried out according to four items of taste, aroma, texture, and color, with a maximum score of 25 points for each item and a total of 100 points. The scoring results were taken as the average value. Specific standards are presented in Table S1.

#### 2.7.3. Lipid and Protein Oxidation Assay

The extent of lipid peroxidation in the minced meat samples was determined using thiobarbituric acid reactive substances (TBARS) as an indicator. Minced meat samples (2 g) were mixed with 18 mL of physiological saline and blended at 8000× *g* for 1 min. Then, 0.2 mL of the resulting mixture was transferred into a 10 mL centrifuge tube. Next, 0.2 mL of 8.1% sodium dodecyl sulphate (SDS) solution, 1.5 mL of 20% acetic acid buffer, 1.5 mL of 0.8% thiobarbituric acid (TBA) aqueous solution, and 0.6 mL of deionized water were added and thoroughly mixed by swirling. Subsequently, the mixture was immersed in a water bath at 95 °C for 1 h, cooled to room temperature, and then centrifuged at 1700× *g* for 5 min. The supernatant was retained and the absorbance at a wavelength of 532 nm was measured. The 1,1,3,3-tetraethoxypropane standard curve was used to calculate the results, which were expressed as mg MDA/kg samples.

The protein oxidation degree of the minced meat samples was assessed by the content of carbonyl and sulfhydryl groups. The carbonyl content was determined by referring to the 2,4-dinitrophenylhydrazine (DNPH) method [25]. The content of sulfhydryl group was determined according to the Ellman method [26].

#### 2.7.4. Analysis of Volatile Flavor Compounds in the Chicken Mince by GC-MS

The volatile flavor compounds of the sample were analyzed using HS-SPME-GC-MS (7890A-5975C GC-MS, Agilent Tech, Santa Clara, CA, USA). First, 1 g of sample was weighed and transferred into a 15 mL extraction vial, which was then quickly sealed. The sample vial was placed on a solid-phase micro-extraction (SPME) device (Supelco, Bellefonte, PA, USA). The temperature was set at 70 °C, and the sample vial was pre-heated on the extraction device for 15 min. The SPME fiber was inserted through the cap into the headspace of the sample for headspace extraction for 40 min. Subsequently, the fiber was inserted into the injection port of the GC-MS system and desorbed at 250 °C for 3 min for injection and analysis. The chromatographic column used was DB-WAX (30.0 m × 250 μm, 0.25 μm). The initial temperature was 40 °C and held for 2 min, then increased at a rate of 5 °C/min to 120 °C, and further increased at a rate of 10 °C/min to 240 °C and held for 5 min. The injection port temperature was 250 °C; the transfer line temperature was 240 °C; and the carrier gas was helium with a flow rate of 1.0 mL/min in a splitless mode. For the mass spectrometry conditions, an EI source was employed with an electron energy of 70 eV. The ion source temperature was 230 °C; and the quadrupole temperature was 150 °C. The scanning mode was Scan, and the scanning mass range was 20–500 u.

### 2.8. Statistical Analysis

All experiments were conducted three times, and the data were expressed as a mean (n = 3) ± standard deviation (SD). All data were statistically analyzed using Duncan’s test and one-way ANOVA of SPSS v. 25.0 (IBM, Armonk, NY, USA). The level of significance was set at *p* < 0.05.

## 3. Results and Discussion

### 3.1. Effects of PEP on the Gel Properties of the Composite Gels

#### 3.1.1. Effects of PEP on the WHC and Cooking Yield of the Composite Gels

**Figure 1:**
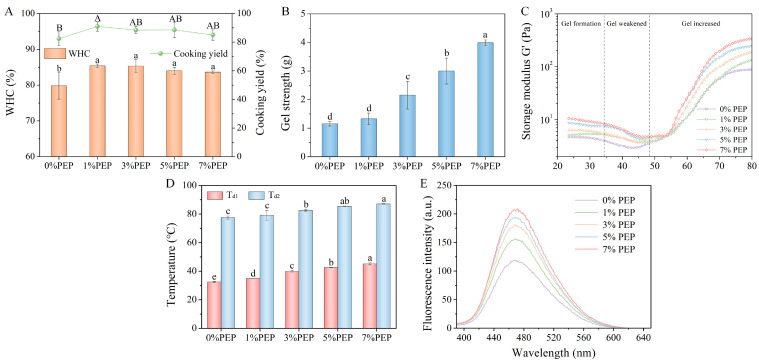
Effect of PEP incorporation on gel properties of the composite gel: (**A**) WHC and cooking yield; (**B**) gel strength; (**C**) storage modulus (G′); (**D**) thermal denaturation temperatures; (**E**) surface hydrophobicity of samples. Different letters indicate a significant difference (*p* < 0.05).

A high WHC and cooking yield are generally associated with superior meat quality, as they can contribute to a more desirable mouthfeel and reduction of shrinkage during cooking [27]. A higher WHC generally represents a robust cross-linking capability between protein and water molecules, leading to the formation of a more consistent and compact three-dimensional network structure within the composite protein gels. As shown in Figure 1A, the MP gel had a WHC of 79.81% and a cooking yield of 82.47%. The incorporation of PEP resulted in a significantly higher WHC and cooking yield of the composite protein gel (*p* < 0.05). These results indicated that the incorporation of PEP may alter the interactions within the gel matrix and water molecules, which further enhances the capacity of the gel to retain water, thereby leading to an enhancement of the WHC. Moreover, the proper incorporation of PEP can significantly reduce the water loss in composite protein gels and increase the cooking yield. This improvement can be ascribed to the fact that, during the gelation process, PEP has extensive interactions with MP, resulting in the denaturation and aggregation of both PEP and MP. This interaction may largely restrict the movement of water molecules, creating a tight three-dimensional network to enhance the retention of water, thereby increasing the cooking yield.

#### 3.1.2. Effects of PEP on the Textural Properties of the Composite Gels

Gel strength is an important metric for evaluating the quality of protein gels, and can reflect the robustness of their internal structure. A higher gel strength implies a more tightly knit gel structure and therefore the formation of stronger gels [28]. As depicted in Figure 1B, there were noticeable increases in the gel strength of the composite gels with an increasing concentration of PEP from 1% to 7%, indicating that a higher concentration of PEP substantially improves the textural attributes of the gel. In general, the above results suggested that the integration of PEP with MP can enhance the stability and integrity of the overall gel network, culminating in a more compact and robust gel structure. Additionally, an increase in the gel strength contributes to the formation of a denser network within the gel, resulting in the encapsulation of more water, thereby enhancing the WHC.

#### 3.1.3. Effects of PEP on the Dynamic Rheology of the Composite Gels

The design, development, and stability prediction of meat products are greatly influenced by their viscoelastic properties [29]. These properties play important roles in determining the texture, mouthfeel, and overall quality of meat products. G′ signifies the elastic component of the viscoelastic behavior, and can delineate the solid-state attributes of the sample. A higher value of G′ indicates stronger ability for the protein to form a gel [11]. As depicted in Figure 1C, all samples exhibited analogous gelation curve features during the heating process. The G′ curve can be primarily divided into three stages, including the gel-forming stage, the gel-weakening stage, and the gel-strengthening stage [30]. In the temperature range of 20–30 °C, the curve tended to be flattened, which signifies the incipient formation of a gel network. At this stage, myosin is cross-linked via interactions between the head-dimers, gradually establishing a more resilient gel network architecture. With increasing temperature, the G′ value started to show a gradual decline, reaching its nadir between 42 °C and 48 °C. At this juncture, the myosin tails are unfolded, and the gel–protein network structure is preliminarily established [31]. Subsequently, upon further heating to 70 °C, the G′ value started to surge dramatically. The high temperature induces complete denaturation of myosin and actin, resulting in an irreversible and highly viscoelastic gel structure, which represents the gel-strengthening stage [32].

Upon the incorporation of PEP, there was a right-ward shift of the minimum value on the G′ curve, indicating that PEP incorporation can increase the temperature at which myosin tails are denatured during the gel-formation process. When the temperature reached 70 °C, the G′ value increased along with the increment of the PEP content, which can be attributed to the fact that a high temperature promotes the unfolding of the protein and the exposure of polar groups, thereby enhancing interactions within the composite gel, giving rise to an elastic and stable gel configuration. Therefore, the incorporation of PEP can remarkably improve the rheological properties of the composite gel.

#### 3.1.4. Effects of PEP on the Thermal Denaturation of the Composite Gels

Protein denaturation is an important step in the formation of the heat-induced MP–PEP gel. Differential scanning calorimetry (DSC) analysis can offer a precise depiction of the conformational shifts that protein molecules undergo during the heat denaturation process [33]. Depending on the incorporation amount of PEP, the MP–PEP composite gels exhibited different thermal behaviors, as shown in Figure 1D. The MP gel mainly had two endothermic transitions, with peak temperatures (T_d_) at 32.46 °C and 77.62 °C, respectively. These two endothermic transitions are due to the thermal denaturation of myosin and actin, respectively [27]. The denaturation temperature of the MP–PEP composite gels increased with the increase of PEP, as indicated by the increase in the T_d_ of myosin and actin. The increase in the denaturation temperature could be attributed to the strengthened interactions between PEP and MP, facilitated by hydrophobic interactions and the formation of disulfide bonds. These interactions lead to tighter binding of the protein molecules, resulting in a more stable and organized protein network structure. This structure is more difficult to be disrupted by thermal energy, resulting in an improved thermal stability of the composite gel. It is also possible that the incorporation of PEP causes the adjustment and rearrangement of the composite gel structure, and the newly formed gel structure is more stable and requires a higher temperature to be disrupted. The high thermal stability of the composite gels offers numerous advantages in the food industry. For example, it can extend the shelf-life of food products by maintaining their structure and functionality, as well as preventing spoilage under high-temperature conditions. It can also help preserve the elasticity and toughness of the composite gels during cooking. This property can enhance the texture and quality of meat products.

#### 3.1.5. Effects of PEP on the Surface Hydrophobicity of the Composite Gels

The surface hydrophobicity of the proteins play a crucial role in maintaining the protein structures, influencing their interactions and specific functional activities. Therefore, the impact of PEP incorporation on the surface hydrophobicity of the MP–PEP composite gels was investigated (Figure 1E). The results showed that surface hydrophobicity increased with the incorporation of PEP. It has been demonstrated that the heating process leads to the exposure of hydrophobic groups that are typically embedded within the structure of the protein [34,35]. The incorporation of PEP may promote the unfolding and expansion of the MP structure during heating, which in turn enhances the overall hydrophobicity.

### 3.2. Effects of PEP on the Structure of the Composite Gels

#### 3.2.1. Changes in Intermolecular Force of the Composite Gels

**Figure 2:**
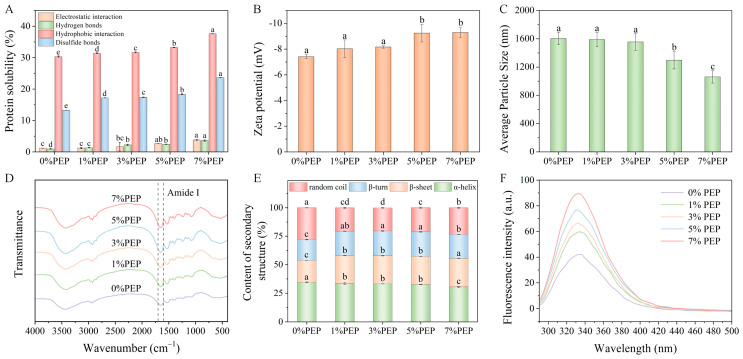
Effect of PEP incorporation on the structure of composite gel: (**A**) intermolecular force; (**B**) zeta potential; (**C**) average particle size; (**D**) FTIR spectra; (**E**) the content of each secondary structure; (**F**) endogenous tryptophan fluorescence spectra. Different letters indicate a significant difference (*p* < 0.05).

The primary forces to construct and maintain the gel structure include ionic bonds, hydrogen bonds, disulfide bonds, and hydrophobic interactions. As shown in Figure 2A, with increasing the incorporation amount of PEP, all four forces in the gel exhibited a continuous upward trend. Compared with the MP gel, the incorporation of 7% PEP only increased the ionic bonds and hydrogen bonds in the MP–PEP composite gel by 2.62% and 2.65%, respectively. This is because during the gel formation process of myosin, its molecules are connected by ionic bonds and hydrogen bonds at the tail. However, heating causes the unfolding of the super-helical structure at the tail, resulting in relatively weak ionic and hydrogen bond interactions. The incorporation of 7% PEP increased disulfide bonds and hydrophobic interactions by 10.37% and 7.23%, respectively. Since heating leads to the denaturation of MP, more hydrophobic groups, sulfhydryl groups, and non-polar amino acids are exposed, which promotes the rearrangement of the hydrophobic regions within the protein and increases the hydrophobicity on the protein surface and its hydrophobic interactions. The exposure of sulfhydryl groups facilitates the polymerization of the MP heads, forming disulfide bonds that play a crucial role in the construction of a gel network [36]. These findings indicate that the incorporation of PEP can promote the unfolding of the internal structure of the MP, thus leading to the formation of a more uniform and dense gel network structure.

#### 3.2.2. Changes in Zeta Potential and Average Particle Size of the Composite Gels

The zeta potential denotes the effective charge of the shear plane surrounding charged particles within a sample system. It is extensively utilized to characterize the interactions among colloidal particles, providing insights into their stability and aggregation tendency [27]. Generally, a lower absolute value of the zeta potential indicates a higher possibility of aggregation and flocculation of the protein particles. Conversely, a higher absolute value of the zeta potential signifies a greater electrostatic repulsion between the particles, leading to a more stable sample system, a finer particle size, and a more uniform distribution [37]. The influence of PEP incorporation on the zeta potential and average particle size of the MP–PEP composite gel is depicted in Figure 2B,C. With an increasing amount of PEP incorporation, the absolute value of the zeta potential exhibited an upward trend. Notably, at the incorporation amount of 5% and 7%, there was a significant rise in the absolute zeta potential and, correspondingly, a significant decline in the average particle size (*p* < 0.05). This could be attributed to the interactions between PEP and MP through hydrogen bonding and hydrophobic forces, which modify the MP structure and lead to the exposure of charged groups and an increase in the surface charge of the composite gel.

However, at lower levels of PEP incorporation, there was no significant change in the zeta potential and particle size, probably because the main chemical force maintaining stability in the composite gel is not hydrogen bonding, which is in line with the results of the intermolecular force analysis mentioned in Section 3.2.1. In addition, PEP is negatively charged at neutral pH. In this study, the pH value was set at 7.0 for protein extraction. The whole system was negatively charged, and the same charges repelled each other. These results indicated that there is electrostatic repulsion between PEP and MP molecules, which enhances the steric hindrance effect between them. In summary, a higher absolute zeta potential coupled with a smaller particle size is indicative of higher stability and more uniform distribution of the composite gel.

#### 3.2.3. Effects of PEP on the Secondary Structure of the Complex Gels

FT-IR spectroscopy was utilized to examine the changes in the molecular structure of MP following the incorporation of PEP. As shown in Figure 2D, the incorporation of PEP did not introduce any new peaks within the 4000–400 cm^−1^ range, but led to variations in the intensity of the characteristic peaks, implying that the interaction between PEP and MP is not mediated by the formation of new covalent bonds but possibly by non-covalent interactions.

The amide I band located between 1700 and 1600 cm^−1^ serves as a key indicator for assessing the secondary structure of proteins. In the context of FTIR analysis, the relationship between the secondary structures and the amide I band is typically characterized by specific wavenumber ranges corresponding to different structural elements. Specifically, the bands within the ranges of 1610–1640 cm^−1^, 1640–1650 cm^−1^, 1650–1660 cm^−1^, and 1660–1700 cm^−1^ correspond to the β-sheet, random coil, α-helix, and β-turn structures, respectively, and the Gaussian function can be employed for curve fitting to accurately delineate these components [38]. The α-helix structure is characterized by orderly arrangement, while the β-sheet and β-turn structures are also relatively ordered. By contrast, the random coil represents a more disordered conformation [39]. The tightness of the protein molecules can be reflected by the total content of α-helix, β-sheet, and β-turn structures, and can also represent the hydrogen bonds between the protein molecules [40].

Figure 2E presents the results of the relative content of the protein secondary structures. It can be observed that the incorporation of PEP markedly influenced the secondary structure of the MP, with α-helix and β-turn as the predominant secondary structural elements in the composition of the composite gels. With an increase in the amount of PEP incorporation from 1% to 7%, the α-helix structure was reduced progressively by 3.30%, 3.85%, 5.59%, and 11.64%, respectively. Concurrently, the random coil structure showed decreases of 26.45%, 27.10%, 24.05%, and 16.24%, respectively. These results indicated that PEP incorporation leads to notable changes in the secondary structure of MP, which may affect the compactness and intermolecular hydrogen bonding of the protein. The β-sheet and β-turn structures exhibited an upward trend. At the incorporation of 7% PEP, the β-sheet content peaked at a maximum of 25.08%, while the β-turn content reached the peak of 21.76% at 3% PEP incorporation. The reduction in α-helix content suggests protein unfolding, and the rise in β-sheet content indicates strengthened protein interactions [4]. The above results suggested that the incorporation of PEP into MP triggers the transition from α-helix to β-sheet structures, enhancing the conformational state and intermolecular interactions, which subsequently augments the gel strength and viscoelastic attributes of the composite gel [41]. The observed increase in β-turn content could be attributed to the rotation of peptide chains, which leads to a more disordered and fragmented network structure compared with the original state [42]. In general, the incorporation of PEP led to a noticeable shift in the structural composition of the MP–PEP composite gels. There were decreases in the content of both the ordered and disordered structures, whereas there were increases in the content of the relatively ordered structure. This trend indicates that during gelation, the helical tail structure of the MP underwent unfolding, and the interactions between the MP and PEP molecules were intensified.

#### 3.2.4. Effects of PEP on the Tertiary Structure of the Complex Gels

Fluorescence intensity measurement can indicate the exposure extent of tryptophan, tyrosine, and phenylalanine to the aqueous environment, and can reflect the changes in the tertiary conformation of proteins [43]. The fluorescence spectra of the composite gels with varying proportions of PEP are depicted in Figure 2F. In general, when the absorption wavelength of the characteristic peak in the fluorescence spectrum of proteins exceeds 330 nm, it indicates that tryptophan is located in a non-polar environment. Conversely, when the absorption wavelength is less than 330 nm, it suggests that tryptophan is located in a polar environment. The above results have shown that the incorporation of PEP to MP enhances the electrostatic interaction between protein molecules, leading to the exposure of amino acid groups in the side chain to a polar environment. With increasing PEP incorporation, there was a slight blue shift of λ_max_, indicating weakening of the polarity in the entire system. Moreover, the maximum emission intensity of the MP–PEP composite gels was significantly higher than that of the MP gel. This is because the hydrophobic interior of the protein was opened up after the addition of PEP, resulting in exposure of more tryptophan on the surface and an expansion of the MP structure. As a result, the hydrophobic environment of the gel system was enhanced. The interaction between MP and PEP leads to an increase in the intensity of the protein reactive fluorescence.

#### 3.2.5. Effects of PEP on the Microstructure of the Composite Gels

**Figure 3:**
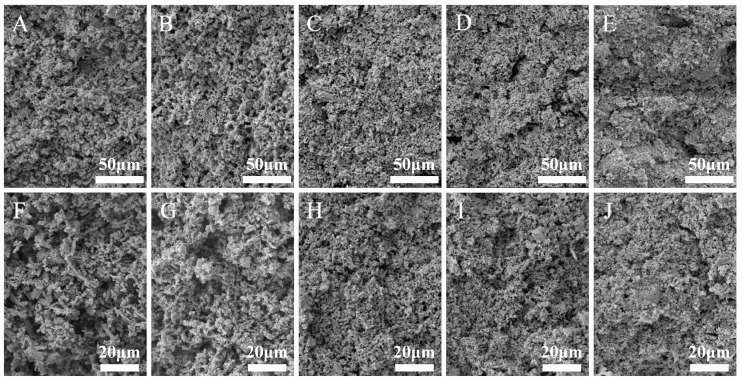
Microstructure of MP–PEP composite gel with 0% (**A**,**F**), 1% (**B**,**G**), 3% (**C**,**H**), 5% (**D**,**I**), and 7% (**E**,**J**) PEP incorporated, respectively.

The scanning electron microscopy images for the microstructure of the MP–PEP composite gels with varying proportions of PEP are depicted in Figure 3. The MP gel was characterized by large pores, distinct fractures between proteins, and a disorganized arrangement. However, with an increase in the incorporation of PEP from 1% to 3%, PEP was observed to fill the pores of the gel, resulting in a matrix with diminished porosity, more delicate cross-linking, a regular shape, and a consistent distribution. When the PEP incorporation was further increased to 5% and 7%, respectively, the gel network structure was partially compromised, presenting a rough appearance with large and irregular holes due to the excessive aggregation of PEP and MP, along with the formation of a substantial amount of the MP aggregates. Overall, an appropriate incorporation of PEP improved the microstructure of the MP–PEP composite gel, leading to a uniform, porous structure with a relatively dense and continuous phase and a pronounced three-dimensional network.

### 3.3. Possible Influencing Mechanisms of PEP on the Composite Gels

Based on the above results, the mechanisms for the action of PEP on the composite gel can be summarized as follows. Upon heat treatment, the embedded chemical groups in MP, such as hydrophobic groups, sulfhydryl groups, and hydrogen bond groups, are exposed, which triggers the aggregation of denatured proteins and leads to the formation of gel networks. However, the MP gel networks are relatively weak and characterized by a large, irregular, and interconnected porous structure. Upon the incorporation of PEP molecules, there were increases in β-sheet and β-turn structures within the composite gel. The PEP molecules effectively fill the voids within the gel and help to connect the MP molecules. This action reduces the size of the initially large voids and leads to the formation of a gel network that is predominantly stabilized through hydrophobic interactions and disulfide bonds. As a result, the gel network within the MP–PEP composite gel becomes more compact and evenly distributed, resulting in enhancement of the overall gel strength and the WHC.

### 3.4. Effect of the P. eryngii Powder on the Quality of the Chicken Mince

The results in Section 3.1 and Section 3.2 have indicated that the incorporation of PEP in specific quantities showed various positive effects on the gel properties of the chicken MP. Therefore, we developed a new type of *P. eryngii* chicken mince product, and explored the influence of the incorporation of the *P. eryngii* powder on the quality and flavor of the chicken mince.

**Figure 4:**
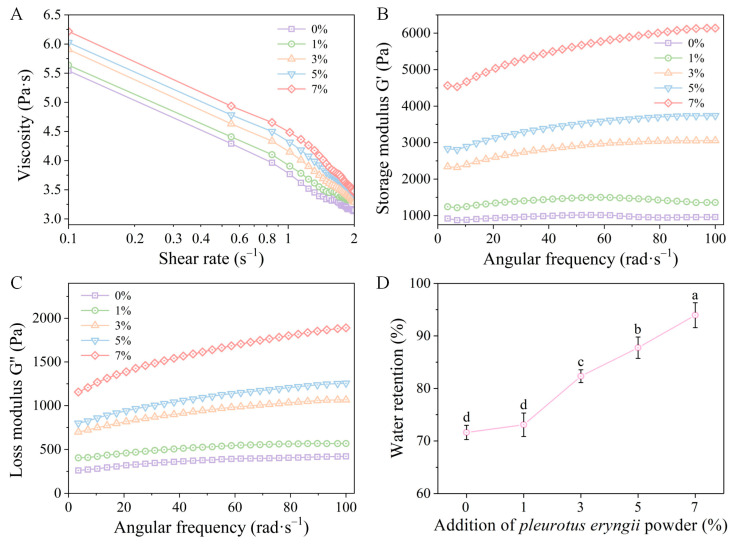
Rheological characteristics of the products: (**A**) viscosity; (**B**) the storage modulus (G′); (**C**) the loss modulus (G″); (**D**) effect of the *P. eryngii* powder on water retention of the chicken mince. Different letters indicate a significant difference (*p* < 0.05).

The rheological properties directly influence the flowability, processability, and palatability of the meat products. As depicted in Figure 4A, the incorporation of the *P. eryngii* powder into the chicken mince resulted in no significant change in the viscosity curve, manifesting a linear viscoelastic region at a shear rate of 1%. With an increase in the shear rate, there was a precipitous decline in viscosity, which is indicative of a pronounced shear-thinning behavior of the minced meat. Moreover, an increment in the proportion of the *P. eryngii* powder resulted in progressive increases in the viscosity of the chicken mince. Figure 4B,C reveal that the G′ of the chicken mince significantly surpasses the loss modulus (G″), suggesting that the rheological characteristics are dominated by elasticity, which is indicative of a robust gel property and the propensity to form an elastic gel network. With an increase in the incorporation of the *P. eryngii* powder, both the G′ and G″ of the chicken mince exhibited an upward trend, indicating that the incorporation of the *P. eryngii* powder can greatly enhance the elasticity and viscosity of the chicken mince. Collectively, the incorporation of the *P. eryngii* powder can exert a significant influence on the rheological attributes of the chicken mince, increasing its viscosity and storage modulus, and concurrently resulting in more pronounced elastic characteristics during frequency sweeps.

WHC is one of the important parameters to evaluate the quality of the minced meat products, which directly affects the flavor, succulence, and tenderness of the product. Figure 4D clearly shows that the incorporation of the *P. eryngii* powder notably enhanced the WHC of the chicken mince in a concentration-dependent manner, and the WHC reached the maximum (93.95%) at the incorporation amount of 7%. This result is in line with the results regarding the WHC of the MP–PEP composite gels, suggesting that the incorporation of the *P. eryngii* powder can improve the quality of the chicken mince.

Coloration is a significant sensory measure of the quality of meat products. As shown in Table 1, incorporation of the *P. eryngii* powder to the chicken mince led to noticeable changes in color metrics. Specifically, there were decreases in the L* value (lightness), while increases in a* and b* values (red–green and yellow–blue color, respectively), and these changes together led to a reduction of the whiteness value. These observed changes can be attributed to the inherent light-yellow color of the *P. eryngii* powder, which reduces the whiteness of the chicken mince upon incorporation. Furthermore, the powder can form an opaque solution upon hydration, which can diminish the effect of light scattering and lead to a lower perceived whiteness when mixed with the mince. In general, the incorporation of the *P. eryngii* powder tends to decrease the brightness of the chicken mince and increase the red and yellow color.

The impact of the *P. eryngii* powder on the texture of the chicken mince is depicted in Table 1. With increasing incorporation of the *P. eryngii* powder, there were obvious increases in the hardness and chewiness of the mince, with less pronounced changes in elasticity, stickiness, and cohesiveness. At the incorporation amount of 3%, the mince exhibited the maximum chewiness and stickiness. At the incorporation amount of 7%, the mince exhibited the maximum hardness, elasticity, and cohesiveness. This trend may stem from the protein in the *P. eryngii* powder, which can enhance the hardness and chewiness by forming a gel structure. However, excessive incorporation of the powder can lead to over-hydration, causing increased hardness and reduced juiciness. Therefore, to balance the taste and texture, the optimal incorporation amount of the *P. eryngii* powder should be controlled between 3% and 5%.

The incorporation of the *P. eryngii* powder showed a significant impact on the sensory quality of the chicken mince (Table 1). When the incorporation amount did not exceed 3%, the sensory score increased with an increasing incorporation of the *P. eryngii* powder, and the texture of the mince became more delicate, along with an improvement of its mouthfeel. The sensory score reached its peak when the incorporation amount was 3%. However, when the incorporation amount exceeded 3%, the sensory score began to decrease, accompanied by varying degrees of decline in the mouthfeel and texture. Therefore, these results indicated that the appropriate incorporation amount of the *P. eryngii* powder is about 3%.

**Figure 5:**
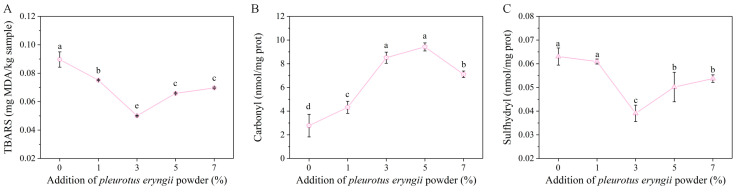
Effect of the *P. eryngii* powder on lipid oxidation and protein oxidation of the chicken mince: (**A**) TBARS; (**B**) carbonyl; (**C**) sulfhydryl. Different letters indicate a significant difference (*p* < 0.05).

Fat oxidation plays an important role in meat flavor development, but excessive oxidation can degrade the taste and quality of the meat products. TBARS value is a common measure of fat oxidation levels in meat. Figure 5A shows that the incorporation of the *P. eryngii* powder to the chicken mince significantly reduced the TBARS value. This reduction can be attributed to the presence of antioxidants in the *P. eryngii* powder, which can slow down the process of fat oxidation, thereby reducing the aldehydes that produce TBARS. The lowest TBARS value was observed at a 3% incorporation amount of the *P. eryngii* powder.

The carbonyl group is the oxidation product of the protein molecules, and the sulfhydryl group can be converted into the carbonyl group during oxidation. Therefore, the carbonyl content can be used as an important index to indicate the degree of protein oxidation. Figure 5B shows that the incorporation of the *P. eryngii* powder to the chicken mince significantly increased the carbonyl content (*p* < 0.05), with the highest level being observed at the 3% and 5% incorporation amount. Measuring the content of the sulfhydryl group can help to evaluate the degree of protein oxidation in meat products, so as to determine its freshness and quality. As shown in Figure 5C, compared with the mince without the *P. eryngii* powder, the *P. eryngii* powder incorporation decreased the sulfhydryl content of the mince, and resulted in the lowest sulfhydryl content (0.0391 mmol/g protein) at the incorporation amount of 3%.

### 3.5. Effect of the P. eryngii Powder on the Flavor of the Chicken Mince

**Figure 6:**
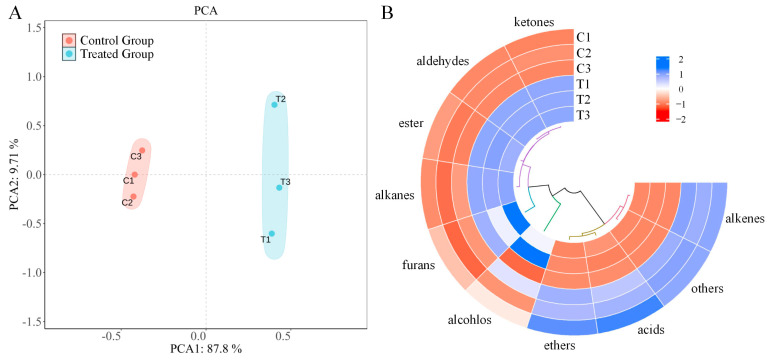
Results of GC-MS analysis on flavor compounds of the *P. eryngii* chicken mince: (**A**) PCA analysis of volatile flavor matter content of different products; (**B**) heatmap of the volatile aromatic substances contents in each sample. Note: C and T in the Figure represent the chicken mince and the *P. eryngii* chicken mince, respectively.

GC-MS was employed to analyze and identify the flavor compounds in the chicken mince with and without the *P. eryngii* powder (Figure S1). A total of 106 volatile flavor compounds were identified. To further elucidate the differences in the volatile flavor compounds between the two groups of samples, a principal component analysis (PCA) was conducted on the composition and content of the volatile flavor compounds (Figure 6A). The results showed that PC1 (87.8%) and PC2 (9.7%) together accounted for 97.5% of the total variance. The samples were found to be within the 95% confidence interval, indicating the reliability of the model and its effectiveness in reflecting the overall sample information. There was a distinct difference in the distribution of the chicken mince with and without *P. eryngii*. The chicken mince without *P. eryngii* was situated in the second and third quadrants, whereas the mince with *P. eryngii* was located in the first and fourth quadrants. The primary differences between the two sample sets were observed along the direction of PC1.

Each volatile compound was quantified and normalized to generate a heatmap (Figure 6B). The heatmap shows that the incorporation of *P. eryngii* into the chicken mince increased the total content of ester, aldehyde, alkane, ketone, and furan compounds, while it reduced alcohol, acid, alkene, ether, and other compounds. These results imply that incorporation of the *P. eryngii* powder endows the chicken mince with a distinct flavor profile. In the absence of the *P. eryngii* powder, the chicken mince contained 70 distinct flavor compounds, comprising 5 acids, 6 aldehydes, 3 esters, 10 alcohols, 29 olefins, 7 alkanes, 4 ketones, 1 ether, 1 furan, and 4 other compounds. Upon the incorporation of the *P. eryngii* powder, a total of 76 flavor compounds were identified, including 4 acids, 5 aldehydes, 4 esters, 13 alcohols, 22 alkenes, 15 alkanes, 7 ketones, 1 ether, 2 furans, and 3 other compounds (Table S2). The principal aromatic compounds within the chicken mince were classified into acids, alkenes, ethers, and other compounds. Notably, the compounds with a substantial contribution encompassed terpinolene, limonene, trans-caryophyllene, α-pinene, γ-muurolene, α-curcumene, estragole, and cis-anethol. In the chicken mince with *P. eryngii*, the predominant flavor compounds were aldehydes, esters, alkanes, and ketones. The compounds with a pronounced contribution included octanal, hexanal, nonanal, pentanol, Oct-1-en-3-ol, and 3,4-epoxy-3-ethyl-2-butanone. Notably, the incorporation of the *P. eryngii* powder to the chicken mince markedly altered the concentration of the predominant flavor compounds and led to the emergence of a novel compound mushroom alcohol. Mushroom alcohol is characterized by the distinctive flavor of *P. eryngii* and is classified as an aliphatic unsaturated alcohol. The increase in these volatile flavor compounds suggests that the incorporation of *P. eryngii* can significantly enhance the flavor complexity of the chicken mince.

Overall, as a sustainable protein source, *P. eryngii* holds great promise in meat processing. Its application can help reduce the reliance on traditional animal proteins and simultaneously open up new markets for fungal proteins. Regarding production costs, *P. eryngii* is a common edible fungus with a wide cultivation range. The costs associated with its production mainly encompass raw materials, such as cultivation substrates, labor, and energy for control of the growth environment. In recent years, advancements in cultivation techniques have led to a continuous increase in the yield of *P. eryngii*, effectively reducing the unit production cost. For instance, the utilization of optimized cultivation substrates and efficient inoculation methods can enhance the growth rate and yield of *P. eryngii*. In terms of processing costs, with the development of drying technologies, like vacuum drying and freeze-drying, although the initial investment in equipment is relatively high, the drying efficiency has been significantly improved. This allows for a reduction in the cost per unit product during large-scale production. From the perspective of product value, *P. eryngii* is rich in essential proteins and other nutrients. Incorporating it into meat products can enhance the quality of the final products, enabling meat-processing enterprises to explore new market segments and expand their market share. For example, by improving gel properties and the WHC, the incorporation of *P. eryngii* can be utilized to develop low-fat, high-protein healthy meat alternatives, meeting the demands of consumers for healthy food. Moreover, incorporating *P. eryngii* to traditional meat products (such as sausages and meatballs) can enhance product texture and WHC, thereby extending the shelf-life and improving the taste. In conclusion, while the production and processing costs of *P. eryngii* need to be taken into account, the potential economic benefits derived from improving product quality, reducing production losses, and expanding market share render the new products economically justifiable.

## 4. Conclusions

This study examined the impact of PEP on the properties of the MP–PEP composite gels and developed a chicken mince product with *P. eryngii*. The main finding is that the incorporation of PEP significantly improves the properties of the MP–PEP composite gels. PEP incorporation optimizes the WHC and cooking yield. The MP–PEP composite gels have more disulfide bonds and hydrophobic interactions, which are important for the quality of the gels. For the chicken mince, the incorporation of 3% *P. eryngii* powder confers the best quality. These results have broad implications for the meat processing industry, and provide a new way to enhance meat product quality with *P. eryngii* and reduce the use of traditional additives.

However, applying these findings industrially faces challenges. Current lab-based experiments must transition to large-scale production. This requires optimizing the production process, such as developing efficient, cost-effective PEP extraction methods. Ensuring the uniform mixing of PEP and MP and maintaining the expected properties of the composite gel is also crucial. Regarding regulations, clear standards for *P. eryngii* use in meat products, including maximum allowable levels and quality specifications, should be set to ensure safety and compliance. Since it changes the product composition, label requirements need re-evaluation. Cost-effectiveness matters in industrial applications. The cultivation and processing costs of *P. eryngii* must be calculated, and cost-reduction strategies must be explored. This can enhance product competitiveness and profitability. In summary, although the present research has yielded valuable results, further research and practical endeavors are required in aspects such as technical improvement, regulatory compliance, and cost control to render industrial application feasible.

## Figures and Tables

**Table 1:** Effects of the *P. eryngii* powder on the color and texture of the chicken mince.

Incorporation Amount (%)	0%	1%	3%	5%	7%
L*	75.62 ± 0.24 ^a^	70.64 ± 0.61 ^b^	63.63 ± 1.28 ^c^	59.31 ± 1.13 ^d^	56.22 ± 2.13 ^e^
a*	−0.54 ± 0.02 ^d^	0.76 ± 0.12 ^c^	1.15 ± 0.42 ^b^	1.73 ± 0.24 ^a^	2.07 ± 0.36 ^a^
b*	9.51 ± 0.10 ^b^	12.18 ± 0.26 ^a^	12.18 ± 0.30 ^a^	12.47 ± 0.32 ^a^	12.60 ± 0.55 ^a^
Whiteness	73.82 ± 0.25 ^a^	68.20 ± 0.64 ^b^	61.63 ± 1.25 ^c^	57.41 ± 1.08 ^d^	54.39 ± 1.95 ^e^
Hardness/N	6.33 ± 0.92 ^c^	8.43 ± 1.47 ^c^	13.29 ± 1.95 ^b^	14.90 ± 2.48 ^b^	17.75 ± 4.49 ^a^
Springiness/mm	5.56 ± 1.24 ^b^	5.71 ± 0.19 ^b^	6.43 ± 0.87 ^b^	6.43 ± 1.24 ^b^	9.37 ± 1.01 ^a^
Cohesion	0.60 ± 0.14 ^a^	0.58 ± 0.13 ^a^	0.57 ± 0.17 ^a^	0.43 ± 0.13 ^b^	0.41 ± 0.15 ^b^
Chewiness/mJ	12.81 ± 5.86 ^c^	18.91 ± 6.59 ^bc^	29.20 ± 11.81 ^a^	22.83 ± 13.20 ^ab^	25.77 ± 9.29 ^ab^
Gumminess/mJ	3.77 ± 1.00 ^b^	4.85 ± 1.08 ^b^	7.36 ± 1.71 ^a^	6.36 ± 1.97 ^a^	6.76 ± 1.44 ^a^
Sensory evaluation score	80.30 ± 1.89 ^cd^	87.10 ± 2.38 ^b^	89.80 ± 2.25 ^a^	82.30 ± 2.75 ^c^	78.20 ± 3.32 ^d^

Note: L*, lightness; a*, redness; b*, yellowness. The data are presented as a mean ± standard deviations (n = 3). Different letters in the same line indicate significant differences (*p* < 0.05).

## Data Availability

The data presented in this study are available in the article.

## Supplementary Materials

The following supporting information can be downloaded at: https://www.mdpi.com/article/10.3390/foods14050752/s1, Figure S1: GC-MS total ion flow diagram of volatile compounds (A: Chicken Mince; B: P. eryngii chicken mince); Table S1: Sensory evaluation criteria; Table S2: Identification of volatile compounds in chicken mince based on GC-MS.
