# Supplementary material for: Influence of Pleurotus eryngii Protein on Myofibrillar Protein Gelation and Application in Chicken Mince Products
Source: Foods. 2025 Feb 23;14(5):752. doi: 10.3390/foods14050752 (PMC11899181; doi:10.3390/foods14050752)
Supplement: Supplementary file 1 [file foods-14-00752-s001.zip › foods-3466157-supplementary.pdf]

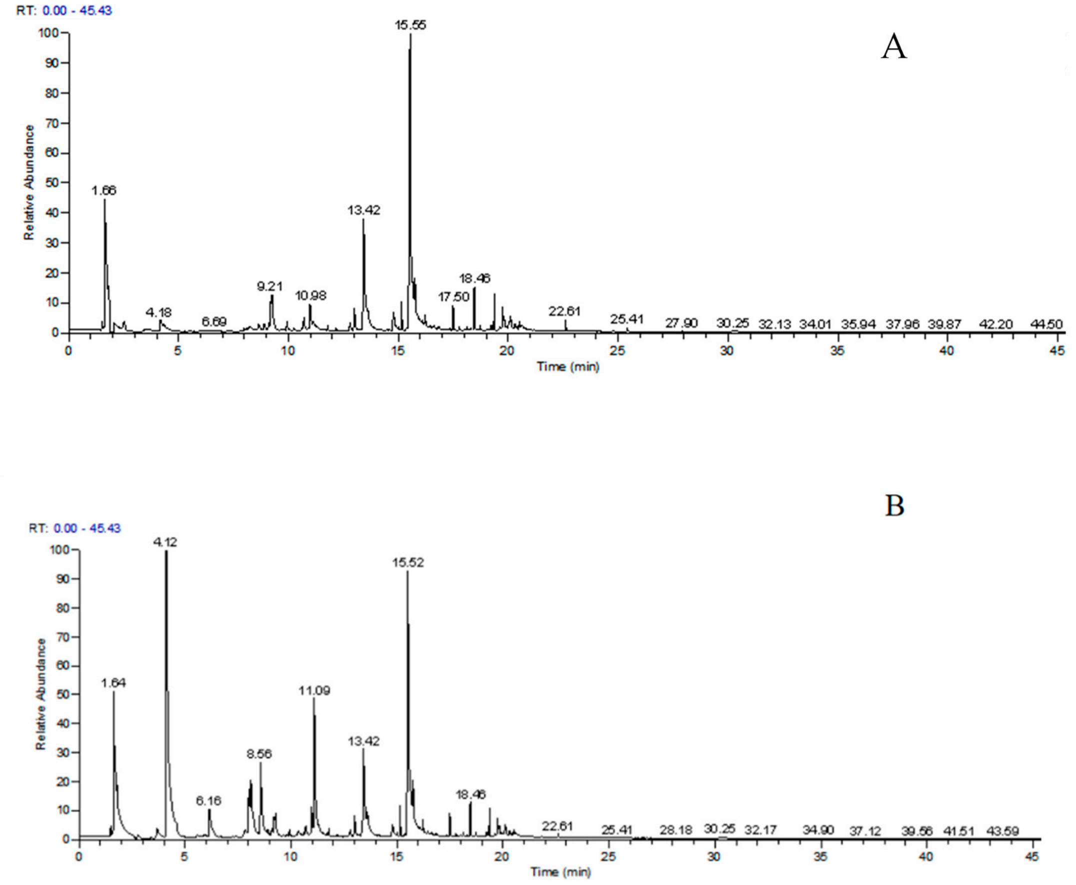

Figure S1. GC-MS total ion flow diagram of volatile compounds (A: Chicken Mince; B: *P. eryngii* chicken mince)

Table S1 Sensory evaluation criteria

Sensory index	Standard for evaluation	Score
Taste and aroma	The meat aroma and the taste of *Pleurotus eryngii* are appropriate, the aroma is harmonious, there are no off-flavors, and the taste is delicious.	20-25
	The meat aroma is strong, slightly with the taste of *Pleurotus eryngii*, the aroma is relatively harmonious, and there are no off-flavors.	15-19
	There is no taste of *Pleurotus eryngii* or it is too strong, and there are off-flavors.	＜15
Mouthfeel	The meat is tender and moderately soft, the mouthfeel is delicate and even, and it has good chewing performance.	20-25
	The meat is slightly dry, the mouthfeel is slightly rough, and the chewing performance is average.	15-19
	The meat is tough, the mouthfeel is rough and uneven, and the chewing performance is poor.	＜15
Color	The minced meat is milky white or light yellow, with bright and shiny color.	20-25
	The minced meat is dark yellow, with distinct color and slightly shiny.	15-19
	The minced meat is yellow-brown, with dull color and no	＜15
Texture	The content is evenly distributed, the texture is elastic, and there are no lumps.	20-25
	The texture is dense, with slight lumps, and poor palatability.	15-19
	The texture is loose, with obvious lumps, and very poor palatability.	＜15

Table S2 Identification of volatile compounds in chicken mince based on GC-MS

NO.		Name	Aroma description	Relative amount (%)	
				C	T
**Acids**	1	Propanoic acid,3-amino-3-oxo-	-	0.03	0.02
	2	2-Methylbenzoic acid	spicy	0.12	-
	3	Pterin-6-carboxylic acid	-	0.09	0.11
	4	(E)-3,7-Dimethyl-2,6-octadienoic acid	-	0.44	-
	5	8-methylnon-6-enoic acid	-	-	0.02
	6	Eicosapentaenoic acid	oily	0.05	0.02
**Aldehydes**	1	Valeraldehyde	-	0.05	0.72
	2	Hexanal	fruity	0.91	17.54
	3	Methyl Valeraldehyde	fruity	0.12	-
	4	Pentanal, 2,4-dimethyl-	-	0.08	-
	5	Heptanal	oily	0.1	1.85
	6	Octanal	-	-	4.55
	7	Nonanal	oily	0.15	8.08
**Esters**	1	3,6-Octadecadiynoic acid, methyl ester	-	0.11	-
	2	2,2,2-trifluoroethyl dodecanoate	-	0.21	-
	3	Bornyl acetate	minty	0.05	0.02
	4	Terpinyl acetate	piney	-	0.21
	5	Terpineol, acetate	resinous	-	1.00
	6	methyl arachidonate	-	-	0.02
**Alcohols**	1	2-ethylhexane-1-thiol	alliaceous	0.17	-
	2	2-Chlorocyclohexanol	-	-	0.05
	3	3,7-Dimethyl-2,6-octadien-1-ol	citrus flavor	0.50	-
	4	pentanol	fruity	-	0.93
	5	1-Hexanol	fruity	-	0.18
	6	3-Tetradecoxypropane-1,2-diol	-	-	0.02
	7	Heptan-1-ol	oily	-	0.63
	8	Oct-1-en-3-ol	mushroom flavor	-	3.43
	9	Cineole	herbal flavor	2.19	1.36
	10	4-Isopropenyl-1-methylcyclohexanol	-	-	0.07
	11	Terpineol	resinous	0.18	-
	12	Linalool	fruity	3.59	1.07
	13	(2Z,5Z)-2,5-pentadecadien-1-ol	herbal flavor	-	0.51
	14	Terpin Monohydrate	-	0.07	-
	15	4-Methoxybenzyl alcohol	fennel flavor	0.71	-
	16	borneol	-	0.66	0.51
	17	(-)-Terpinen-4-ol	citrus flavor	2.03	1.24
	18	5-(4-fluoroanilino)-3H-1,3,4-thiadiazole-2-thione	allium flavor	0.02	0.02
**Alkene**	1	5-vinyl-2-norbornene	-	0.05	-
	2	cuparene	-	0.09	-
	3	(+)-α-pinene	resinous	1.11	0.06

	4	Bicyclo[3.1.0]hex-2-ene,4-	-	-	0.06
	5	Camphene	spicy	0.26	-
	6	Myrcene	-	0.66	-
	7	α-phellandrene	vanilla flavor	0.66	-
	8	Terpinene	citrus flavor	-	0.33
	9	Terpinolene	citrus flavor	1.69	0.13
	10	1,5,5-trimethyl-3-methylidenecyclohexene	-	-	0.12
	11	limonene	lemon flavor	5.84	-
	12	(+)-Limonene	citrus flavor	5.19	-
	13	trans-Anethole	fennel flavor	0.12	0.17
	14	2-ethylbutyl methacrylate	-	-	0.88
	15	2,5-dimethyl-3-vinyl-1,4-hexadiene	-	0.1	-
	16	(4Z,6Z,9Z)-1,4,6,9-Nonadecatetraene	-	0.19	0.11
	17	trans-Caryophyllene	flower scent	3.56	1.83
	18	Alpha-pinene	pine scent	1.9	1.11
	19	α-bergamotene	lemon flavor	0.27	0.36
	20	(1R,3Z,9S)-4,11,11-trimethyl-8-methylenebicyclo[7.2.0]undeca-3-ene	-	0.22	0.04
	21	α-caryophyllene	-	0.44	0.24
	22	γ-Muurolene	-	1.59	1.42
	23	α-Curcumene	-	1.76	1.53
	24	α-Ylangene	herbal flavor	0.08	1.01
	25	Curcumene	-	0.42	-
	26	α-Bisabolene	spicy	0.44	0.47
	27	Amorphous poly alpha olefin	-	0.21	-
	28	cedrene	plant scent	0.21	-
	29	2,4-Hexadiene	fruity	0.52	-
	30	3-(1,5-Dimethyl-4-hexenyl)-6-methylenecyclohexene	-	0.59	-
	31	cubane	-	0.04	-
	32	α-calacorene	spicy	0.08	0.1
	33	Caryophyllene oxide	-	0.03	0.04
	34	4,6-Decadiene	-	-	0.18
	35	4,10-Dioxo-3,6,9,12-tetrahydro-3,6,9,12-tetramethyl-1,4,7,10,13-pentacyclo [6.3.3.0] tetradeca-3,6,9,12-tetraene	-	-	0.02
	36	trans-Sesquisabinene hydrate	resinous	-	0.03
**Alkane**	1	Decane	-	0.03	0.18
	2	4-(1-Methylethylidene)bicyclo[0.7.1]heptane	-	-	0.11
	3	3-(Trifluoroacetoxy)pentadecane	-	-	0.21
	4	2-(Trifluoroacetoxy)tridecane	-	-	0.04
	5	tetrakis(trifluoroacethoxy)silane	-	-	0.28
	6	6-methyloctadecane	-	0.3	0.21
	7	2,6,10-Trimethyldodecane	-	0.98	1.60

	8	2,6,10-Trimethylpentadecane	-	0.08	0.05
	9	2,6,11-Trimethyldodecane	-	-	0.06
	10	2,7-Dimethyloctane	-	-	0.04
	11	pentadecane	-	0.70	0.68
	12	cyclohexane	-	0.24	0.25
	13	Tetradecane	-	0.14	0.15
	14	2-(7-Heptadecynyloxy)tetrahydro-2H-pyran	-	-	0.05
	15	4,7-Dimethylundecane	-	-	0.26
**Ketones**	1	4-Methyl-2-hexanone	fruity	-	0.21
	2	2-Heptanone	creamy flavor	-	0.21
	3	1-Bromo-3-phenylpropan-1-one	-	0.06	-
	4	2,6-Di(4-Azidobenzal)-4-Methylcyclohexanone	vanilla flavor	-	0.59
	5	benzylidene camphor	-	1.1	0.50
	6	Camphor	mint flavor	0.22	-
	7	3,4-Epoxy-3-ethyl-2-butanone	-	-	8.22
	8	3,6-dimethyloctan-2-one	creamy flavor	-	0.03
	9	2,2-dimethylcyclohexanone	-	0.03	-
	10	(+/-)-Camphor	woody scent	-	0.02
**Ethers**	1	Estragole	-	9.70	5.13
**Furans**	1	3,6,7-Trimethoxy-1(3H)-isobenzofuranone	fruity	-	0.09
	2	3,4,6-trimethoxy-3H-2-benzofuran-1-one	-	0.15	0.10
**Others**	1	Toluene	-	0.02	-
	2	p-Xylene	oily	0.04	-
	3	1-methyl-2-propan-2-ylbenzene	-	0.46	0.26
	4	cis-Anethol	fennel flavor	38.85	20.11
	5	(-)-Isoborneolaceticacid	mint flavor	-	0.11
